# Viruses, bacteria and parasites: infection of the male genital tract and fertility

**DOI:** 10.1186/s12610-023-00193-z

**Published:** 2023-07-20

**Authors:** Rachel Guiton, Joël R. Drevet

**Affiliations:** Université Clermont Auvergne, CNRS UMR6293, GReD Institute, 63001 Clermont-Ferrand, France

**Keywords:** Male genital tract, Infertility, Infections, Viruses, Bacteria, Parasites, Tractus génital masculin, Infertilité, Infections, Virus, Bactéries, Parasites

## Abstract

**Background:**

Infertility affects one couple out of six worldwide. Male infertilty can result from congenital or acquired factors, of which pathogens that reach the genital tract through sexual contact or blood dissemination. The impact of major viral, bacterial and parasitic infections on the male genital tract and fertility has been summarized.

**Results and conclusions:**

A systematic review of articles published in the Google Scholar and PubMed databases was conducted. It turns out that viruses, as well as bacteria and parasites are major inducers of male genital tract infections and ensuing infertility through damage to the organs and subsequent loss of function and/or through direct damage to the sperm cells. Moreover, not only male infertility results from such infections but these can also be transmitted to women and even to the offspring, thus highlighting the need to efficiently detect, treat and prevent them.

## Résumé

**Contexte:**

L’infertilité affecte un couple sur six dans le monde. L’infertilité masculine peut être due à des facteurs congénitaux ou acquis, parmi lesquels des pathogènes qui atteignent le tractus génital par contact sexuel ou dissémination par voie sanguine. Cette revue présente les principaux pathogènes d’origine virale, bactérienne et parasitaire qui affectent le tractus génital masculin et leur impact sur la fertilité.

**Résultats et conclusion:**

Une revue systématique de la littérature a été conduite à partir de Google Scholar et de PubMed. Il apparaît que les virus, au même titre que les bactéries ou les parasites, sont des facteurs majeurs d’infection du tractus génital masculin et d’infertilité. Cette dernière découle de dommages aux organes reproducteurs et à leur perte de fonction et/ou d’atteintes directes aux spermatozoïdes. De plus, ces infections n’impactent pas seulement la fertilité masculine, mais elles peuvent également être transmises aux partenaires féminines et même à la descendance, ce qui souligne l’importance de les détecter, de les traiter et de les prévenir efficacement.

## Introduction

Up to one in six couples fails to achieve pregnancy within one year of unprotected intercourse, and a male-associated infertility is found in half of these childless couples. Male infertility can result from congenital or acquired urogenital abnormalities, genetic disorders, endocrine disturbances, malignancies or immunological factors, some of which may be the consequence of urogenital tract infections [1]. According to the World Health Organization (WHO), more than 30 viruses, bacteria, and parasites are transmitted through sexual contact. Eight of these pathogens are responsible for sexually transmitted diseases presenting the greatest incidences worldwide: *Hepatitis B Virus*, *Herpes Simplex Virus*, *Human Immunodeficiency Virus*, *Human Papillomavirus*, *Treponema pallidum*, *Neisseria gonorrhoeae*, *Chlamydia trachomatis* and *Trichomonas vaginalis* [2]. However, infertility can also originate from blood-borne pathogens like *Hepatitis B Virus* or commensal organisms like *Escherichia coli* or *Staphylococcus aureus*. Some studies point to an incidence of 6 to 18% of infertile men presenting with genital tract infections [3, 4], but this incidence is quite hard to appreciate as a result of differences in the definition of infertility and in the regions of the world that are considered in the studies [5, 6].

The present review is not meant to be exhaustive, neither regarding the pathogens nor the literature which is huge for some aspects, but rather focuses on pathogens that actually affect the male genital tract or sperm and that are known to or highly suspected to cause subsequent infertility. To this end, human studies are exclusively mentioned here, even if animal models are available for most of the included pathogens and give clues to explain the mechanisms of human physiopathology which are sometimes uneasy to understand due to hardly accessible human samples.

## Viruses

Viruses can reach genital organs through sexual contact or via the systemic route. However, some of them do not damage the male genital tract organs but rather impact sperm cells, thus resulting in infertility (Table 1).

**Table 1:** Major viruses infecting men and their consequences on male fertility

Viruses	Infected genital organs	Damage to genital tract organs	Causes of male infertility
*Hepatitis B virus*	• Seminal fluid and spermatozoa [7–9] • Testis [10, 11]	• Testicular pain [12] • Testicular vasculitis and thrombosis [12] • Scrotal swelling and pain [13, 14] • Epididymitis [13, 14]	• Lower sperm quality [15–20]
*Herpes Simplex virus*	• Penis [21, 22] • Urethra [21, 23] • Testis [24] • Expressed prostatic secretions [25] • Prostate tumour tissue [26] • Seminal vesicles and semen [4, 24, 27–29] • Spermatozoa [30]	• Urethritis and urethral discharge [21, 23, 31] • Epididymo-orchitis [32] • Penile vesicles and lesions [21–23] • Local pain and itching [23] • Dysuria [21, 23] • Chronic prostatitis [25] • Controversial association with prostate cancer [26, 33, 34]	• Controversial effects on sperm parameters [4, 27–30] *however:* • Possible impaired prostatic and epididymal functions [4]
*Human Immunodeficiency Virus*	• Penis [35–37] • Urethra [36, 37] • Prostate [38, 39] • Seminal vesicles and semen [40, 41] • Testis [38, 39, 42] • Epididymis [39]	• Erosion of seminiferous tubule epithelium [39] • Necrosis of seminiferous tubule walls [39] • Testicular interstitial fibrosis [43] • Thickening of seminiferous tubule basement membranes [43] • Epididymal obstruction [39, 43] • Testicular cancer [44]	• Impaired spermatogenesis [39, 43] • Azoospermia [39] • Lower sperm counts and ejaculate volumes [45, 46] • Fewer progressively motile sperm [45–47] • Alteration of sperm morphology [46]
*Human Papillomavirus*	• Penis [48] • Urethra [48, 49] • Testis [50] • Epididymis [51] • Vas deferens [52] • Prostate [53, 54] • Semen [48, 52, 55–57]	• Penile cancer [49] • Prostate cancer, controversial [58, 59] • Prostate disturbances [55]	• Lower chances of pregnancies [60–62] • Higher risks of miscarriage [55, 60, 61] • Low sperm morphology score [60, 61] • Increased sperm DNA fragmentation index [61] • Decreased sperm progressive motility, controversial [55–57, 60, 61]
*Mumps virus*	• Testis [63] • Semen [64]	• Scrotal swelling and pain [65, 66] • Hydrocele [65] • Epididymo-orchitis [65] • Orchitis [63, 64, 66–68] • Testicular atrophy [66–68]	• Decreased serum testosterone level [66, 67] • Impotency [67] • Decreased sperm count and motility [64, 69] • Altered sperm morphology [64, 69] • Oligozoospermia and azoospermia [64, 66, 68, 69] • Hypofertility or infertility?
*SARS-CoV-2*	• Testis [70, 71] • Semen [72–76]	• Decreased testis volume [77] • Orchitis [71, 78] • Degeneration/loss of germ cells and spermatocytes [71, 78, 79] • Sertoli cells alteration [78] • Thickening of seminiferous tubule basement membranes [78, 80] • Increased apoptotic testicular cells [71, 79] • Testicular microthrombosis [78] • Epididymitis [81, 82] • Scrotal and testicular pain [82] • Possible prostatic infarction and acute urinary retention [83]	• Not directly reported *however:* • Impaired spermatogenesis and oligozoospermia [71, 78, 79] • Decreased total testosterone level [77, 84–86] • Decreased seminal volume, sperm count, sperm concentration and total sperm motility [77, 79, 86]

This table summarizes the organ location of the main viruses that are known to infect the male genital tract. It also specifies the damage to these organs and the proven or possible causes of male infertility as reported in the literature

*DNA* deoxyribonucleic acid, *SARS-CoV-2* severe acute respiratory syndrome coronavirus 2

### Hepatitis B virus

*Hepatitis B virus* (HBV) infection is a viral liver infection which can cause both acute and chronic diseases. It is a major global health problem as hepatitis B resulted in more than 800,000 deaths (mostly from cirrhosis and liver cancer) in 2019, while almost 300 million people were chronically infected. Despite the fact that an efficient and safe vaccine is available, 1.5 million new infections are still observed each year [87]. Another risk for infected people was highlighted by a population-based study that showed a statistically higher risk of infertility in men with HBV infection [88]. The exact causes of infertility are not understood yet but a significantly lower sperm quality has been described in infected men when compared to uninfected men, in a context of infertility or not. Affected sperm parameters were diverse and encompassed motility, viability, and morphology [15–19]. Some studies directly identified HBV DNA in seminal fluid and sperm cells [7, 8], and even visualized the HBV DNA sequence integration into the sperm chromosomes, leading to increased chromosome aberrations [9, 20]. Thus, even if no negative impact on the success of assisted reproductive therapies was seen [16, 17], worries can be reasonably raised about the long term consequences on the progeny’s health. HBV nucleic acids have also been found in testicles, and more precisely, in intertubular stromal fibroblasts on samples obtained from patients who died of either acute or chronic hepatitis B [10, 11]. A rare complication of hepatitis B called polyarteritis nodosa, which is a necrotizing vasculitis affecting medium-sized arteries, has also been described in a 35-year old man presenting with long lasting testicular pain, and this was associated with thrombosis [12]. On the other hand, although no direct identification of HBV was done in epididymal samples, this organ is affected as epididymitis have been described during acute HBV infections [13, 14]. Finally, the association of HBV infection with extrahepatic cancers has long been questioned but different recent large-scale studies could refute this idea [89, 90].

### Herpes Simplex Virus

Herpes, caused by *Herpes Simplex Virus* (HSV) infections, is a common and global pathology. Two types of HSV infect humans. HSV type 1 (HSV-1), which is generally transmitted by oral-to-oral contact, is responsible for orofacial mucosal surfaces infections (oral herpes) although it can also cause genital herpes. HSV type 2 (HSV-2), which is mainly sexually transmitted, causes genital herpes that affects more than 49 million people aged 15–49 years worldwide [91]. Both HSV infections can become recurrent although it is more common with HSV-2 than with HSV-1. HSV-1 genital infections, although less frequent than HSV-2 infections, can cause urethritis and urethral discharge, as well as epididymo-orchitis which is a rare manifestation of HSV-1 infection observed in a patient presenting a severe sepsis [31, 32]. HSV-2 has been shown to infect penis and urethra, causing urethritis and penile vesicles, but it also infects testis [21, 23, 24]. A large-cohort study revealed that pain, itching, dysuria and urethral discharge were the main local symptoms observed following primary HSV-2 infections. These were the same symptoms during secondary infections but the proportion of men presenting with dysuria and urethral discharge decreased [23]. Moreover, Bowman et al. described a rare manifestation of HSV-2 infection as penile verrucous lesions that are almost exclusively seen in immunocompromised patients, and especially *Human Immunodeficiency Virus* (HIV)-positive patients [22]. HSV-2 DNA has also been described in expressed prostatic secretions of men suffering from chronic prostatitis [25]. The association between HSV infection and prostate cancer is still controversial. Indeed, some studies could not find any association between antibodies to HSV-2 and prostate cancer [33, 34] while a recent work demonstrated an increased expression of two *herpes virus*-encoded miRNAs in prostate tumour tissues, which could be observed in benign prostatic hyperplasia cells [26]. Finally, the fact that HSV infection induces male infertility is also controversial as some studies mention possibly impaired prostatic and epididymal functions in infected men [4], or decreased sperm density and motility, as well as low sperm counts [4, 27, 30] while others did not [28, 29].

### Human Immunodeficiency Virus

*Human Immunodeficiency Virus* (HIV) is still a global major public health issue as 38 million people were living with HIV in 2021, mostly in Africa. HIV infection is not curable even if the disease can be managed by a combination of antiretroviral drugs, making this infection a chronic health condition. Semen and vaginal secretions are the predominant means of contamination, however people under efficient antiretroviral therapies (ART) do not transmit HIV to their partners. Of note, global ART coverage was 75% in 2021, according to the WHO [92]. The final stage of HIV infection is acquired immunodeficiency syndrome (AIDS), which can take years to develop if not treated. Many pathologies of the male genital tract resulting from HIV infection have been described thanks to AIDS patients. Interventional studies confirmed observational studies that predicted male infection through the penis, as male circumcision provided a protection of 60% against HIV infection [35]. Other studies identified foreskin Langerhans cells and urethral macrophages as HIV targets and/or reservoirs [36, 37]. However, not only immune cells can be infected by HIV but also spermatogonia, spermatocytes and spermatids [38, 42]. In fact, almost all of the organs/tissues of the male genital tract can be targeted by HIV, such as the prostate, seminal vesicles, testis and epididymis [38–40, 42]. HIV infection causes damage to infection sites, such as erosion of seminiferous tubules epithelium and necrosis of seminiferous tubules walls [39], fibrosis of testicular interstitium [43], and epididymal obstruction [39, 43], leading to impaired spermatogenesis and even azoospermia [39, 43]. Moreover, the presence of HIV in sperm has been associated with poor semen parameters, especially fewer progressively motile spermatozoa [45–47]. HIV-infected patients also often present with hypogonadism, which is a well-known cause of infertility [93]. Interestingly, a recent study proposed that this condition could be partly compensated by the use of combined ART [94]. Finally, some studies suggested a possible association between HIV infection and testicular cancer [44].

### Human papillomavirus

*Human papillomavirus* (HPV) infection is one of the most common viral sexually transmitted diseases worldwide. Among the five genera of HPVs, *Alphapapillomaviruses* (alpha HPVs) are widely associated with genital tract infections, and can be classified into three types. The high-risk HPVs are the etiological agents of multiple cancers (HPV16 is the most oncogenic of the high-risk HPVs). The low-risk HPVs (of which the HPV6 and HPV11) are generally associated with genital warts. Finally, the cutaneous HPVs cause common and plantar warts [95]. HPV DNA from both high-risk and low-risk viruses has been identified in the whole male genital tract, from the penis [48] and urethra [48, 49] to the testis [50], epididymis [51], and vas deferens [52], and even to the prostate [53, 54]. A study established that HPV persistence in penile samples was significantly higher than in semen samples, and that oncogenic genital HPVs were more likely to persist for 6 months or longer than non-oncogenic HPVs [48]. In correlation with this result, an association between HPV infection and penile cancer has been proven [49], which is still not the case for prostate cancer, a point of controversy [58, 59]. Even if no tissue damage has been described, the presence of HPV in male partners is highly associated with lower chances of ongoing pregnancies and a higher risk of miscarriage [55, 60–62]. Moreover, a meta-analysis showed that the prevalence of HPV in semen is higher in infertile men than in the general population [61]. Infertility more likely results from altered sperm parameters, as characterized in numerous studies: low sperm morphology score [60, 61] and increased sperm DNA fragmentation index [61]. However, a decrease in sperm progressive motility is still controversial [55–57, 60, 61]. A recent study on 151 infertile couples proved that anti-HPV vaccination could significantly improve sperm progressive motility and anti-sperm antibodies, while increasing the chances of positive pregnancy outcomes [62].

### Mumps virus

Mumps virus (MuV) is the causative agent of mumps, a disease that was first described by Hippocrates in the fifth century before Christ, but its viral etiology was only demonstrated in the 1930s [96]. MuV is only known to infect humans and spreads via direct contact or by airborne droplets from the upper respiratory tract of infected patients. Mumps is generally self-limiting and as so, not a life-threatening disease which disappears without sequelae. Nonetheless, some people can suffer from well-known complications of mumps, of which orchitis or epididymo-orchitis [65, 97]. Even if MuV can only be detected in early samples of orchitis [63], many studies suggest long-term testicular damage, in particular testicular atrophy [66–68]. In addition to reproductive organs damage, rare cases of gynecomastia have been described, developing after 1 to 42 years of mumps orchitis and testicular atrophy. This breast development was associated with low serum concentrations of testosterone, suggesting a long-term and progressive loss of function of Leydig cells. Two of the three men mentioned in this study had no more children after developing mumps orchitis [67]. A secondary hypogonadism has also been described in a young man during the acute phase of mumps [66]. On the other hand, MuV RNA has been isolated in the seminal fluid collected from mumps patients [64], and long-term declines in sperm quality were demonstrated. Indeed, studies showed damage to sperm count, motility and morphology early after orchitis onset and persistent oligozoospermia or asthenozoospermia even one year later [64, 66, 69]. Still, subsequent male infertility keeps on being controversial as testicular damage is mainly unilateral and some authors suggest that the other testicle may compensate for this one-side loss of function, an assertion supported by the fact that some patients with mumps orchitis can father children [67, 68]. This is why mumps orchitis is generally admitted to cause hypofertility rather than infertility.

Although vaccination has dramatically reduced the incidence of mumps in children, mumps orchitis occurs in approximately 30% of unvaccinated and 6% of vaccinated post-pubertal male patients [98]. Three cases of orchitis that occured following MMR (measles, mumps, and rubella) vaccination were reported in 2010, bringing the total number of cases to 13 at that time [99]. Due to the rapid orchitis onset in two of the three cases, the authors raise the possibility that an immune response was responsible for the testicular inflammation instead of a direct viral invasion and replication, even though they had no biological evidence to support their idea. This study clearly points to the need for a better understanding of mumps pathophysiology.

### SARS-CoV-2

A first case of severe atypical pneumonia, caused by the severe acute respiratory syndrome coronavirus 2 (SARS-CoV-2), was declared in Wuhan, China in december 2019. This disease was then formally named coronavirus disease 2019 (COVID-19) by the WHO. To date the ensuing pandemic has already affected about 640 million people worldwide and caused more than 6.6 million deaths [100]. It is now known that SARS-CoV-2 infection is transmitted through respiratory droplets which penetrate the upper respiratory tract. The initial attachment of SARS-CoV-2 to the host cell is mediated by the binding of the viral spike protein to its receptor, the angiotensin-converting enzyme 2 (ACE2). Host proteases, such as transmembrane serine protease 2 (TMPRSS2), are then needed to cleave the spike protein, allowing permanent fusion of the viral and host cell membranes [101]. Male gender is a well-known risk factor for COVID-19 [102], and ACE2 and TMPRSS2 expressions have been described in testis and prostate, respectively, strongly suggesting that the male genital tract may also be infected by SARS-CoV-2 [103]. Subsequent studies confirmed the presence of SARS-CoV-2 in testis and associated COVID-19 with severe testicular damage, including lower testicular volume, orchitis characterized by a scattered infiltration of immune cells into the interstitial compartments, massive loss or degeneration of germ cells and spermatocytes, swelling and vacuolization of Sertoli cells, thickening of the tubular basal membrane, increased number of apoptotic cells, and microthrombosis in the testicular vasculature [70, 71, 77–80]. Cases of epididymitis have also been mentioned in the literature although without a direct observation of viruses in the epididymis [81, 82]. The reduced spermatogenesis observed in most cases has also been associated with disturbances in sex homones metabolism, in particular decreased total testosterone levels in infected men, which may negatively impact male fertility, especially during the acute phase of the disease [77, 84–86]. However, a lower sperm count does not seem to be the sole consequence on sperm quality as significant reductions of seminal volume, sperm concentration and total sperm motility were also noticed in SARS-CoV-2 patients [77, 79, 86]. Finally, SARS-CoV-2 RNA could not be found in expressed prostatic secretions from COVID-19 patients [104], and its detection in seminal fluid is still controversial. Indeed, about twenty studies could not identify viral RNA in semen samples from infected men (listed in [72, 105]) while five succeeded, both in acute phase samples and after recovery [72–76]. A recent study suggested that the lack of detection in semen samples could result from the use of unsuitable RT-PCR methods, i.e. not validated on such samples [72]. However, as some teams could find SARS-CoV-2 RNA in semen samples, one cannot rule out a possible transmission to the partner.

## Bacteria

Among the eight bacteria that were included in the present review, three are responsible for one of the eight most prevalent sexually transmitted infections worldwide: *Chlamydia trachomatis*, *Neisseria gonorrhoeae* and *Treponema pallidum*. The other ones are known or strongly suspected to have detrimental effects on male fertility: *Escherichia coli*, *Mycobacterium tuberculosis*, *Staphylococcus aureus* and *Mycoplasma* spp (Table 2). As mentioned in the introduction, not all of them were included even if they have proven negative impact on male fertility, such as *Streptococci* or *Helicobacter pylori* but interested readers can refer themselves to a recent review [106].

**Table 2:** Selected bacteria infecting men and their consequences on male fertility

Bacteria	Infected genital organs	Damage to genital tract organs	Causes of male infertility
*Chlamydia trachomatis*	• Urethra [107–109] • Prostate [110, 111] • Epididymis [108, 112] • Testis [113] • Seminal vesicles and semen [111, 114–116]	• Urethritis [107, 117] • Prostatitis [110] • Epididymo-orchitis [107] • Epididymitis [112] • Seminal vesiculitis [114] • Enlarged seminal vesicles [109]	• Controversial [118]
*Escherichia coli*	• Urethra [119] • Prostate [120] • Testis [121, 122] • Epididymis [108] • Seminal vesicles [123–125]	• Prostatitis [120, 126] • Urethritis and urethral discharge [119] • Epididymo-orchitis [119, 121, 122] • Testicular pain and swelling [121] • Testicular infarction [121, 122] • Scotal, testicular, seminal vesicle and prostatic abscesses [121–125]	• Controversial but association with poor sperm parameters [127, 128]
*Mycobacterium tuberculosis*	• Urethra [129, 130] • Prostate [131–135] • Seminal vesicles [132, 136] • Testis [131, 137, 138] • Epididymis [131, 139, 140]	• Urethral fistula [129, 130] • Urethral stricture [129, 130] • Enlarged prostate [129, 131, 133] • Prostatitis [131, 133] • Urethral, prostatic, testicular and epididymal granulomatous lesions [130, 131, 133, 137–139] • Urethral, prostatic, testicular and epididymal necrosis [130–134, 137, 138] • Enlarged seminal vesicles [136] • Prostatic, seminal vesicle and testicular calcifications [131, 136, 137] • Scrotal, prostatic and seminal vesicle abscesses [129, 132, 134, 135] • Enlarged testis [131, 132, 138] • Enlarged epididymis [131, 132, 136, 139] • Epididymitis [129, 139, 140] • Scrotal pain and swelling [137, 138, 140]	• Undefined cause [140] • Azoospermia [129, 136, 141]
*Neisseria gonnorhoeae*	• Urethra [108, 142] • Semen [143, 144]	• Urethritis and urethral discharge [108] • Dysuria [108] • Epididymo-orchitis [142] • Higher prostate cancer risk [145, 146]	• Controversial [106] *however:* • Temporary or permanent oligozoospermia to azoospermia [142]
*Staphylococcus aureus*	• Seminal vesicles and semen [127, 147–156] • Testis [157] • Prostate [125, 151, 158, 159]	• Seminal vesicle, prostatic and testicular abscesses [125, 150, 151, 157, 159, 160] • Epididymo-orchitis [150, 157] • Foci of prostatic and testicular necrosis [157, 160] • Enlarged and tender prostate [125, 150, 151, 158, 160] • Dysuria [125, 150, 151, 157, 158]	• Controversial [148, 152–155]
*Treponema pallidum*	• Semen [161, 162] • Penis [162, 163] • Testis [164–166]	• Penile and scrotal lesions [162] • Scrotal or testicular swelling [163–165, 167–169] • Testicular gummas [165, 167–170] • Atrophy/destruction of seminiferous tubules [164, 168, 170] • Destruction of testicular parenchyma [168] • Testicular obliterative vasculitis [164] • Orchitis [165, 168, 169] • Epididymo-orchitis [164, 166, 167] • Higher prostate cancer risk [146, 171]	• Not directly reported
*Ureaplasma urealyticum*	• Urethra [172] • Semen [173–179] • Expressed prostatic secretions [110, 180] • Epididymis [181]	• Urethritis [182] • Epididymitis [181, 183] • Chronic prostatitis [110, 179, 180] • Possible association with prostate cancer [184]	• Controversial effects on spermatozoa [173–175, 177, 178] • Elevated ROS in semen [179]

This table summarizes the organ location of eight bacteria that are known to infect the male genital tract. It also specifies the damage to these organs and the proven or possible causes of male infertility as reported in the literature

*ROS* reactive oxygen species

### Chlamydia trachomatis

Chlamydial infections are the most common bacterial sexually transmitted infections. Indeed, the WHO estimated that around 130 million people were infected by *Chlamydia trachomatis* in 2020 [2]. This represents a real public health issue as these infections are asymptomatic in approximately 70% of women with well-knowm detrimental effects on their fertility [2]. Fifty percent of men infected with *C. trachomatis* are asymptomatic but others present signs of urethritis [107, 117], prostatitis [110], epididymo-orchitis [107], or epididymitis [112] in association or not with seminal vesiculitis [114]. Nonetheless, the effect of chlamydial infections on men fertility is still controversial [118] as there is no consensus as to the mechanisms affected in men. However, reports showed damaged sperm quality in infected patients, and ex vivo experiments also pointed to detrimental effects on sperm function after a direct contact with *C. trachomatis* [185–187].

### Escherichia coli

*Escherichia coli* strains are the most frequent causes of urinary tract infections but genital tract can also be affected by retrograde ascent. Sometimes these bacteria are acquired through female partners [119]. Extra-intestinal pathogenic *E. coli* (ExPEC), and particularly uropathogenic *E. coli* (UPEC) are known to cause acute prostatitis [120, 126]. In fact, contrary to commensal strains, UPEC have acquired various virulence factors that allow them to colonize mucosal surfaces and to cause damage to host tissues [120]. Male genital tract infection can also lead to urethritis [119], epididymo-orchitis [119, 121, 122], or testicular pain and swelling [119, 121, 122]. Consequences of such infections can be dramatic as some cases of testicular infarction and loss of testicle despite appropriate antibiotic treatment have been described [121, 122]. More rarely, the active infection of the seminal vesicles can lead to the development of abscesses that can be cured by appropriate antibiotics [123, 124]. Interestingly, *E. coli* are among the most frequently isolated bacteria in the semen of infertile men [127, 128, 147, 148]. However, their role in causing male infertility is still debated as some studies significantly associated *E. coli* infection with decreased sperm quality [127, 128] while others did not [148, 149]. Of note, there are growing in vitro evidence that different parts of the bacteria can directly affect sperm function, such as outer membrane vesicles or cell wall components [188–190].

### Mycobacterium tuberculosis

Tuberculosis, which is caused by *Mycobacterium tuberculosis*, is present all over the world but, in 2020, new cases particularly affected the South-East Asian region, the African region, and the Western Pacific region, according to the WHO [191]. Even if tuberculosis is curable and preventable, it is the 13th leading cause of death worldwide and the second deadly leading infectious disease after COVID-19, that is above AIDS. This situation is especially worrying as multi-drug resistance is increasing. By chance, not all infected people will develop the disease but immunodeficient people are at higher risk of falling ill [191]. When bacteria enter the respiratory tract, *M. tuberculosis* can settle in the lungs and begin to grow, before potentially affecting other parts of the body, such as the kidneys, the brain, or the spine [192]. Althought *M. tuberculosis* spreading to the genital tract is controversial, it has been proposed to rely on different mechanisms: hematogenous, retro-urethral, lymphatic, or direct extension [131]. Almost all male genital organs can be affected by the presence of *M. tuberculosis*. In the urethra, it generally causes fistula and/or stricture that require surgical procedures [129, 130]. Many reports describe granulomatous prostatitis [131–133], resulting from the formation of granulomas surrounding areas of caseous necrosis, prostatic calcifications or abscesses [129, 131, 132, 134–136]. Colonization of the seminal vesicles is rarely found but also results in enlargement, calcifications or abscesses [132, 136]. Most of the time, when testis is infected, epididymis also is, even if some cases show isolated tuberculous orchitis or epididymitis [137]. Once infected, these organs usually show granulomas and necrosis [131, 137–140]. Abscesses and granulomas often mislead to a cancer diagnosis that is detrimental to the patient as it delays the appropriate treatment [131, 193]. Finally, male infertility, and more precisely azoospermia, has been associated with genital tuberculosis, often resulting from obstructions [129, 136, 140, 141].

### Neisseria gonorrhoeae

*Neisseria gonorrhoeae* is one of the most prevalent sexually transmitted bacterium. It accounted for 82 million new cases in 2020, mostly in young adults, in Africa and in the Western Pacific region [194]. As for *M. tuberculosis*, it is a real health problem as multi-resistance to currently available antibiotics is constantly increasing [194]. *N. gonorrhoeae* exclusively infects human by entering the mucosal epithelium, of which that of the urogenital tract [195]. Most of men infected with *N. gonorrhoeae* are symptomatic and present with urethral discharge and dysuria, that precede scrotal pain [108, 142, 195]. Epididymo-orchitis has been described in young adult men but surprisingly *N. gonorrhoeae* could not be isolated from aspirated epididymal fluid [108, 142]. The direct effect of gonorrhoea on man fertility is still controversial [106] but recent studies showed that the *N. gonorrhoeae* infection prevalence was significantly higher in an infertile population than in a fertile population [143, 144]. Moreover, testicular histopathology of biopsies from infertile infected men revealed necrosis of seminiferous tubules, heavy infiltration of inflammatory immune cells during the acute phase of infection, associated with temporary or permanent oligozoospermia, and even azoospermia in some patients who completed a two-year follow-up [142]. Finally, *N. gonorrhoeae*-infected men were shown to be at higher risk of developing prostate cancer compared to controls without gonorrhoea [145, 196]*.*

### Staphylococcus aureus

*Staphylococcus aureus* is a Gram-positive bacterium that belongs to the *Micrococcaceae* family. Its name refers to the golden coloration of the colonies. Given the presence of *S. aureus* on various parts of the human body, *i.e.* the nares, vagina, pharynx, skin surface, it is considered a commensal organism. Indeed, it is estimated that 30–50% of healthy adults are colonized by *S. aureus* [197]. However, infections can occur through a breach in the skin or through mucosa, allowing bacteria to disseminate to neighboring tissues or to the bloodstream [197]. *S. aureus* is a major causative agent of skin, respiratory, bone, joint, and soft tissue pathologies, and can even cause lethal endocarditis. The typical manifestation of *S. aureus* infection is abscess formation [197], and such a manifestation has been described in the male genital tract. Prajapati et al. described a very rare case of testicular abscess originating from *S. aureus* infection in a 55-year old Indian man. This abscess formation was associated with epididymo-orchitis. Although the abscess cavity needed to be curetted for necrotic tissue, the follow-up diagnosis revealed minimal inflammatory changes and testis viability [157]. *S. aureus* abscesses in the seminal vesicles are also rarely mentioned in the literature [150, 151]. Less rare are the prostatic abscesses due to *S. aureus* infection. The most prevalent associated symptoms are urinary urgency, frequency, and/or retention, and dysuria [125, 151, 158–160]. Whatever their location, these abscesses were never mentioned to be the source of severe tissue lesions which could induce infertility. In fact, the impact of *S. aureus* on male fertility is still controversial. Indeed, studies identified *S. aureus* in the semen of infertile men, sometimes in association with altered sperm parameters [148, 152–154] but a recent case–control study revealed that the percentages of *S. aureus*-positive semen samples from fertile and infertile men were not statistically different [155]. Finally, the controversy may also lay in the fact that the percentage of semen positive for *S. aureus* highly differs from studies and/or from countries, some showing little sperm contamination [149, 155, 156], others medium contamination [148, 154], and even high contamination [127, 147, 152, 153].

### Treponema pallidum

Although rarely found in semen [161, 162], *Treponema pallidum* is the etiological agent of syphilis, which is one of the four curable sexually transmissible infections with chlamydia, gonorrhoea and trichomoniasis [2]. Syphilis accounted for 7.1 million new cases in 2020 according to the WHO estimates [2]. Early *T. pallidum* infection is well-known to cause chancres (painless ulcers) on various parts of the body, and especially on the penis and scrotum [162, 163]. Among the other male genital tract organs, testis was the only site where the presence of *T. pallidum* has been described in the literature. In addition to the observed testicular and/or scrotal swelling [163–165, 167, 168], the diagnosis often relies on ultrasound examination and sometimes reveals a testicular mass suspicious for malignancy, which generally leads to orchidectomy [165–167], even if some work succeeded in saving the patient’s testicle using antibiotics [163]. Testicular infection can give rise to typical gummatous orchitis [165, 167–170], that is a central necrotic area surrounded by inflammatory cells (mainly lymphocytes and plasma cells) or to atypical lesions devoid of necrosis [164, 166]. Other testicular damage were described, like atrophy or destruction of the seminiferous tubules [164, 168, 170], destruction of parenchyma [168] or obliterative vasculitis in some blood vessels [164], making syphilis a very likely cause of male infertiltiy, even if no infertility cases directly resulting from *T. pallidum* infection have been reported. Finally, like gonorrhoea, syphilis has been linked to an increased risk of developing prostate cancer [146, 171].

### Ureaplasma urealyticum and Mycoplasma genitalium

*Ureaplasma urealyticum* and *Mycoplasma genitalium* are parts of the genus *Mycoplasma* that comprises more than 120 species. *Mycoplasma* spp. are bacteria found in the mouth, and the upper respiratory and urogenital tracts [198]. *U. urealyticum* was first isolated from men with nongonococcal urethritis in the 1950s [182] and since it has also been shown to be part of the natural urethra flora of some healthy men [172]. Apart from the urethra, *U. urealyticum* was identified in semen [173–179], expressed prostatic secretions [180, 199] and epididymal aspirates [181] while being implicated in various inflammatory settings in the male genital tract, *i.e.* epididymitis [181, 183] and chronic prostatitis [179, 180, 199]. A recent meta-analysis of the literature showed that prostate cancer patients have 3.6 times increased odds of being colonized with *Ureaplasma* spp., which suggests a possible association between a chronic infection and prostate cancer [184]. The direct effects of *U. urealyticum* on male fertility are still debated as some studies on infected men demonstrated a significant decrease in the sperm cells quality (motility, vitality, concentration, chromatin condensation, DNA integrity) [177, 178] while others did not [174, 175]. However, Potts et al. showed a higher reactive oxygen species (ROS) level in semen from infected men with chronic prostatitis compared to healthy men, and ROS are well-known lipid peroxidation inducers that could be responsible for decreased membrane fluidity and reduced sperm fertilization capability [179]. *M. genitalium* is a major cause of nongonococcal urethritis in men [200]. However, its relatively low prevalence, which is consistently reported to be around 1% in young men in the United States [201], in Britain [202], as well as in Denmark [203], makes it unlikely to be a common cause of sexually transmitted infection worldwide.

## Parasites

Parasitic infections of the male genital tract are not as frequent as viral and bacterial infections. However, some well-known parasites have shown their ability to reach this compartment, sometimes with severe consequences on fertility. Their so far described location and impact on male fertility is summarized in Table 3.

**Table 3:** Major parasites infecting men and their consequences on male fertility

Parasites	Infected genital organs	Damage to genital tract organs	Causes of male infertility
*Acanthamoeba* spp*.*	• Prostate [204] • Testis [205]	• Necrotic lesions in testicular parenchyma [205] • Fibrin thrombosis of testicular small arteries and capillaries [205]	• Not directly reported
*Entamoeba histolytica*	• Testis [206] • Epididymis [206] • Seminal vesicles and fluid [207] • Prostate [208] • Penis [209]	• Vacuolation of germ cells [206] • Increased and edematous testicular stroma [206] • Erosion of epididymal duct wall by the parasite [206] • Sperm and fibrin clots in epididymal lumen [206] •Dilation of epididymal tubules [206] • Enlarged and tender seminal vesicles [206, 207] • Prostatitis [208] • Ulcers on the prepuce [209]	• Not directly reported *however:* • Aspermatogenesis in many seminiferous tubules [206]
*Leishmania* spp*.*	• Penis (cutaneous form) [210–214] • Testicular macrophages? (visceral form) [215]	• Suspected damage to seminiferous tubules [215]	• Dysfunction of the pituitary–gonadal axis [215] • Suspected deregulation of spermatogenesis [215]
*Plasmodium* spp*.*	• Testis [216, 217]	• Testicular pain and swelling [216, 217]	• Oligozoospermia, necrozoospermia, temporary azoospermia [218] • Lower testosterone levels [219]
*Schistosoma* spp*.*	• Prostate [220, 221] • Seminal vesicles and semen [220, 221] • Testis [220, 221] • Epididymis [220, 221]	• Dyspareunia [220] • Prostatitis [220] • Haematospermia due to ulceration of seminal vesicule mucosal lining [220] • Hydrocele [220] • Orchitis [220] • Epididymitis [220]	• Oligozoospermia, azoospermia [220, 221] • Poor sperm motility and viability [220–222]
*Toxoplasma gondii*	• Testis [223–227] • Prostate [228]	• Epididymitis [224] • Orchitis and testicular necrosis [223, 225–227] • Testicular granulomas [224]	• Not directly reported *however:* • Lower sperm concentration and motility [229] • Anti-sperm antibodies [230]
*Trichomonas vaginalis*	• Urethra [231] • Prostate [231–233] • Testis [234, 235] • Epididymis [233, 236]	• Urethritis [231] • Prostatitis [231–233] • Orchitis [234] • Atrophic testes [234, 235] • Epididymitis [233, 236]	• Low serum testosterone [234, 235] • Hypogonadism and oligoasthenoteratozoospermia [234] • Azoospermia [235, 236] • Decreased spermatozoa motility and morphological alterations [237]
*Trypanosoma brucei*	• No direct organ infection described	• Atrophy of seminiferous tubules [238] • Reduction of testicular volume [239]	• Testicular hypogonadism [240, 241] • Lower testosterone plasma levels [240–242] • Impotency [239, 241–243]
*Trypanosoma cruzi*	• Testis [244–246]	• Epididymo-orchitis [244] • Hypoplasia of germ cells [245, 246] • Maturation arrest of germ cells [245, 246] • Degeneration of Leydig cells [245, 246]	• Not directly reported *however*: • Loss of libido and impotency [245, 246] • Oligozoospermia to azoospermia in chronically infected men [245]
*Wuchereria bancrofti*	• Testis [247, 248]	• Hydrocele [249] • Epididymo-orchitis [247, 249] • Atrophy of seminiferous tubules [250] • Recurrent scrotal pain and swelling [251] • Elephantiasis [250]	• Oligozoospermia and non-motile spermatozoa [249] • Azoospermia [250]

This table summarizes the organ location of the parasites that are known to affect male fertility. It specifies the parasite location in the male genital tract, the damage to these organs and the proven or possible causes of male infertility as reported in the literature. It is of note that even if no direct infection of the genital tract is mentioned in the literature, *Trypanosoma brucei* is clearly associated with possible causes of infertility

*Spp* species plural

### Acanthamoeba spp

*Acanthamoeba* species are ubiquitous protozoans that live in natural habitats. They can be at the origin of a localized and painful keratitis in immunocompetent hosts. However, as opportunistic pathogens often associated with AIDS, they give rise to a systemic and eventually lethal illness in immunocompromised hosts. Only rare reports of their presence in the male genital tract can be found but they were described in the prostate [252] and testis [205] of infected men, the latter being an AIDS patient. Even if no direct effect on fertility is reported in the literature, the necrotic lesions discovered in the testicular parenchyma, as well as the thrombosis of small arteries and capillaries suggest that this infection can lead to severe defects in reproductive functions.

### Entamoeba histolytica

*Entamoeba histolytica* is a worldwide anaerobic protozoan. The infection predominantly involves the gastrointestinal tract and the liver, but some cases of extra-intestinal diseases have been reported. Indeed, this parasite has been identified in almost all organs of the male genital tract. These cases are considered amoebic dysentery with amoebic metastases in the genital tract. In 1922, the first report of a reproductive tract infection by *Entamoeba histolytica* came from Aldred Scott Warthin who showed parasites disseminated throughout the testes and epididymes with marked phagocytosis of spermatozoa by the amoebas in the epididymal and testicular tubules. Moreover, many seminiferous tubules presented with aspermatogenesis and vacuolation of germ cells, and an edematous stroma. In the epididymis, an actual active erosion of the duct wall by the parasites was noted as well as sclerotic blood vessels. Finally, there were fibrinous clots containing amoebas in the vas deferens [206]. Dissemination to the seminal vesicles has been reported one year later, in a young man presenting slightly enlarged and extremely tender seminal vesicles [207]. Along the male genital tract, prostate can also be the site of an *Entamoeba histolytica* infection, as was shown by its identification during urinalysis after prostatic massage on an American patient [208]. Also, one case of penile amoebic infection associated with a carcinoma was described in India, where the histopathological observation of a penile ulcer revealed a large number of *Entamoeba histolytica* parasites in the floor of the ulcer [209]. As for *Acanthamoeba* species, no direct infertility cases were reported following *Entamoeba histolytica* infections but given the tissue destruction that was observed in testis and epididymis, one can expect deleterious effects on the reproductive function of infected men.

### Leishmania spp.

*Leishmania* infections can be divided into cutaneous and visceral forms, the latter being lethal if left untreated, also known as kala-azar. The cutaneous form usually affects uncovered parts of the body in endemic zones. However, if they are extremely rare, lesions on genitals have been described worldwide over the last 20 years. Ulcers or a giant hyperkeratotic form of leishmaniasis on the glans penis on patients from South America [210, 211] or Middle East [212], a nodular ulcerative lesion on the prepuce in association with histiocytes containing numerous *Leishmania* on an Italian patient [213], subcutaneous nodules on the penis on a Tunisian man [214] are some examples found in the literature. No direct effect of kala-azar on the male genital tract has been reported but in a cross-sectional study, the authors strongly suggested deleterious effects on fertility (deregulation of spermatogenesis and damage to seminiferous tubules) secondary to the dysfunction of the pituitary–gonadal axis that they observed [215].

### Plasmodium spp.

Although five *Plasmodium* species are known to infect humans, two of them are of particular interest as *P. falciparum* is the main causative agent of malaria in Africa, and *P. vivax* is responsible for the majority of cases outside of sub-Saharan Africa. Malaria, one of the most common and deadly diseases, is acquired by the bite of infected female mosquitoes and causes an acute febrile illness. In 2020, it was estimated that nearly half of the world’s population was at risk of this parasite disease [253]. Only few reports mention a direct relationship between male genital tract or reproductive capacities and malaria. Two case reports from the late 1980’s outlighted testicular pain and swelling associated with *P. falciparum* [216, 217] while another study described severe sperm defects, in terms of count and morphology [218]. The authors strongly suspected that repeated fever episodes during malaria attacks could affect the highly temperature-sensitive process of spermatogenesis [218]. More recently, a study including eight *P. vivax* infected-men from Honduras showed that they presented lower testosterone levels than healthy controls [219], with a possible impact on both immunity and reproductive functions.

### Schistosoma spp.

Schistosomiasis, also known as bilharzia, is acquired throught skin penetration of blood flukes present in infested water. This pathology is prevalent in tropical and subtropical areas where infected people can develop either an intestinal or a urogenital form of bilharzia, depending on the parasite species. The urogenital form of the disease is mainly caused by *Schistosoma haematobium* which is prevalent in Africa, the Middle East, and Corsica (France) [254]. Some cases of infertility resulting from *S. mansoni* or *S. japonicum* infections have also been reported [222, 255]. The accumulation of parasite eggs in various genital tract organs induces inflammation and tissue damage. A wealth of publications is available in the literature to describe the affected organs and damage leading to infertility due to *Schistosoma* male genital tract infection. Unfortunately it is impossible to cite them all in this review, thus the authors recommend very well-documented and well-written recent reviews which are summarized in Table 3 [220, 221, 256]. Overall, male infertility results from oligozoospermia or azoospermia consecutive either to a testicular or accessory glands dysfunction due to tissue inflammation and granuloma formation or to a post-testicular dysfunction due to obstruction of excurrent ducts. However, despite lots of evidence concerning its severe consequences on fertility, bilharzia is still underestimated due to a lack of diagnosis and surveillance in endemic areas [256].

### Toxoplasma gondii

Toxoplasmosis is a ubiquitous disease which prevalence varies greatly between countries, from 10 to 80%. Moderate and high prevalences are found in Central and Southern Europe, and in Latin America and tropical African countries, respectively [223]. It is a relatively asymptomatic infection in immunocompetent hosts but life-threatening in immunocompromised persons, and especially in AIDS patients who usually develop neurological symptoms due to the reactivation of a latent infection [223]. Only rare studies identified *Toxoplasma gondii* in the male reproductive tract of immunocompetent men. They presented epididymitis and orchitis with necrotising and non-necrotising granulomas [224], or a testicular mass showing extensive fibrosis and necrosis under histological examination [225]. Both patients had testicular parasites. Other studies have reported testicular toxoplasmosis associated with orchitis and tissue necrosis in AIDS patients, as well as prostate toxoplasmosis [223, 226–228]. In these case reports, no direct effect on fertility was demonstrated but various cross-sectional studies point to arguments in favour of a deleterious role of *T. gondii* infection in men, such as lower sperm concentration and motility or higher anti-sperm antibodies in infertile men compared to fertile individuals [229, 230].

### Trichomonas vaginalis

*Trichomonas vaginalis* is the etiological agent of trichomoniasis, the sexually transmitted infection with the most elevated number of new infections worldwide (156 million in 2020) [2]. Although historically associated with non-gonococcal urethritis, the presence of *T. vaginalis* has been demonstrated in various parts of the male genital tract [231–236]. Most of the time, trichomoniasis is asymptomatic in men, which is quite problematic as a study showed that the physical characteristics of the seminal fluid was significantly altered in infected men compared to healthy individuals as was the morphology and motility of sperm cells [237]. As these alterations were corrected in half of the patients by a single course of metronidazole, the reference treatment for *Trichomonas* infections, the authors suggested that the infertilty seen in asymptomatic men may be due to infection by this parasite [237]. Moreover, infertility of men presenting with *T. vaginalis* testicular infections has been described in association with low serum testosterone, as a consequence of atrophic testes associated with low sperm concentration and loss of motility, or azoospermia due to *T. vaginalis* cytotoxicity [234, 235]. Amar also reported cases of epididymitis characterized by swollen and tender epididymis as well as thickened vas deferens [233]. Another case report described the presence of *T. vaginalis* in epididymal aspirates associated with azoospermia resulting from a maturation arrest at the spermatocyte level in both testes [236]. Live *T. vaginalis* have been found in prostatic fluid, and intraepithelial vacuolization of the prostate has been observed, suggesting an active infection of this gland and subsequent painful inflammation [232, 233]. Finally, *T. vaginalis* infection has long been suspected to be associated with prostate cancer risk. However, at least two recent meta-analyses of the literature demonstrated that there was no statistically significant association between a previous *T. vaginalis* exposure and the risk of developing prostate cancer [196, 257]. At the same time, another study found out that there was no correlation between *T. vaginalis* serostatus and mortality among prostate cancer patients [258].

### Trypanosoma brucei

Human African trypanosomiasis, also known as sleeping sickness, is acquired throught the bite of a tsetse fly. This vector, which is only found in sub-Saharan African countries, can transmit *Trypanosoma brucei* parasites [259]. *T. brucei* is divided into 3 major subspecies, two of them being responsible for human diseases: *T. brucei gambiense* (Tbg) at the origin of a chronic disease, and *T. brucei rhodesiense* (Tbr) at the origin of an acute form of the disease. In both cases, the disease is lethal if left untreated. Although no direct infection of the reproductive tract has ever been reported in man, mouse testes are sites of invasion in experimental settings [260] and infertility is a well-known consequence of the human infection. Infected men are often presenting with impotency, loss of libido, lower testosterone levels than healthy individuals [239–243]. A significant atrophy of the seminiferous tubules was also reported in two human cases [238]. It is suggested that the ability of *T. brucei* to interfere with the endocrine functions, and especially the hypothalamic-pituitary–gonadal axis would cause the reproductive dysfunctions in men [241].

### Trypanosoma cruzi

Chagas disease, caused by *Trypanosoma cruzi*, affects 6–7 million people who mainly live in Latin America [261]. Even if male infertility is not especially mentioned in the literature, Chagas, who gave his name to this American trypanosomiasis, identified parasites in the testicles of acutely or chronically infected men often in association with epididymo-orchitis [244]. In addition, hypoplasia or maturation arrest of testicular germ cells, as well as degeneration of Leydig cells, were described in chronic Chagas disease patients. These testicular damage were more pronounced in oligo- and azoospermic men compared to normozoospermic individuals [245, 246]. Finally, infected men often report a loss of libido and/or potency. These symptoms could be due to denervation of the pelvian plexus ganglia which are responsible for innervating the genitals [245].

### Wuchereria bancrofti

*Wuchereria bancrofti* is the main parasite at the origin of lymphatic filariasis, also known as elephantiasis. It is responsible for 90% of the reported cases [262]. More than 860 million people in 47 countries worldwide are at risk of being infected. The disease develops as the adult worms nest in the lymphatic vessels, thus disrupting the normal function of the lymphatic system [262]. The majority of infections is first asymptomatic but when becoming chronic, it often leads to severe damage, especially genital damage as the lymphatics of the spermatic cord are a favoured location. Among the genital manifestations often described are recurrent scrotal pain and swelling, hydrocele, and epididymo-orchitis [247, 249–251]. Parasites have been shown to infect testis [247, 248], and atrophy of the seminiferous tubules in association with azoospermia was seen in an Indian man with long-standing infertility [250]. At least two other studies described oligozoospermia and non-motile spermatozoa in men originating from India and Nigeria [249, 251].

## Conclusions

This review highlights the multitude of pathogens that are able to degrade male fertility, through directly damaging reproductive organs or sperms cells. Despite the huge amount of data available on the topic, it is really hard to determine the main type of pathogens that is associated with male infertility. For example, the viruses as well as the parasites mentioned in this study, affect billions of people worldwide but not all of these infected patients will become infertile [2, 87, 100, 253]. One major issue is that these infections are often asymptomatic and present negative effects on reproductive functions only long after the contamination, thus preventing an undoubtful identification of the etiological agent [66–68, 223, 262]. Another substantial issue comes from the difficulty of establishing direct links between bacterial or viral infections and an actual impact on male fertility.

Moreover, when reading the literature, it is obvious that infections are rarely due to unique pathogens but rather result from co-infections. Some pathogens are well-known for their co-occurrence in patients, such as *C. trachomatis* and *T. vaginalis*, *Mycoplasma*, *N. gonorrhoeae* or HPV [107, 110, 112, 187, 263], or *E. coli* and *T. vaginalis* [110]. Regarding sexually transmitted infections, exposure to one agent is often a predictor of exposure to another one, such as HSV-2 exposure and HIV infection [143]. Moreover, some infections are known to facilitate the pathophysiology of others, even if the mechanisms are not fully understood. One stricking example is that HIV-positive patients are 18 times more likely to develop active tuberculosis than HIV-negative people [92]. The same harmful association exists between *N. gonorrhoeae* or *T. pallidum* and HIV [195, 264].

Another interesting point to consider is that some pathogens, especially viruses, may negatively influence assisted reproductive therapies outcomes [16, 60, 61]. For example, in vitro fertilizations (IVF) performed using sperm from HBV-infected male partners lead to lower fertilization rates although implantation and pregnancy rates are not affected by infection [16]. The situation is quite different when using sperm from HPV-positive men as they induce higher miscarriage rates and lower pregnancy rates following IVF [60, 61]. This could result from the entry of HPV into the sperm cells and subsequent transfer to the oocyte where viral genes are efficiently transcribed [265]. In fact, vertical transmission is a true concern, even during natural conception. For instance, vertical transmission of *T. brucei* has been reported in a 19-month old child from his mother who became infected by her asymptomatic partner [266]. Vertical transmissions have been reported for other pathogens, such as *T. gondii* [267], *C. trachomatis* [111], *N. gonorrhoeae* [195] or viruses [268], sometimes with long-term consequences for the offspring, even on fertility.

Finally, this review points to the need for further studies regarding the immune responses at work in the genital tract, which could explain some cases of male infertility through their deleterious consequences on reproductive organs (*e.g*. mumps orchitis [66, 99]). The main problem is getting access to human specimens infected with non life-threatening pathogens. This is why appropriate animal models are of tremendous interest, even if it is uneasy when it comes to human-specific pathogens such as *N. gonorrhoeae* or MuV.

All these reasons make it urgent to put efforts on controlling the spread and understanding the pathophysiology of the viruses, bacteria and parasites affecting male fertility.

## Data Availability

Not applicable.

## Abbreviations

ACE2: Angiotensin-converting enzyme 2
AIDS: Acquired ImmunoDeficiency Syndrome
ART: Antiretroviral therapies
COVID-19: Coronavirus disease 2019
C. trachomatis: Chlamydia trachomatis
DNA: Deoxyribonucleic acid
E. coli: Escherichia coli
ExPEC: Extraintestinal pathogenic Escherichia coli
HBV: Hepatitis B Virus
HIV: Human Immunodeficiency Virus
HPV: Human Papillomavirus
HSV: Herpes Simplex Virus
IVF: In vitro fertilization
M. genitalium: Mycoplasma genitalium
miRNA: Micro ribonucleic acid
MMR vaccination: Measles, Mumps and Rubella vaccination
M. tuberculosis: Mycobacterium tuberculosis
MuV: Mumps virus
N. gonorrhoeae: Neisseria gonorrhoeae
P. falciparum: Plasmodium falciparum
P. vivax: Plasmodium vivax
RNA: Ribonucleic acid
ROS: Reactive oxygen species
RT-PCR: Reverse transcription-polymerase chain reaction
SARS-CoV-2: Severe acute respiratory syndrome coronavirus 2
S. aureus: Staphylococcus aureus
S. japonicum: Schistosoma japonicum
S. mansoni: Schistosoma mansoni
T. brucei: Trypanosoma brucei
T. gondii: Toxoplasma gondii
TMPRSS2: Transmembrane serine protease 2
T. pallidum: Treponema pallidum
T. vaginalis: Trichomonas vaginalis
U. urealyticum: Ureaplasma urealyticum
WHO: World Health Organization
